# Association between psychosocial functioning, health status and healthcare access of asylum seekers and refugee children: a population-based cross-sectional study in a German federal state

**DOI:** 10.1186/s13034-021-00411-4

**Published:** 2021-10-12

**Authors:** Diogo Costa, Louise Biddle, Kayvan Bozorgmehr

**Affiliations:** 1 Department of Population Medicine and Health Services Research, School of Public Health, Bielefeld University, P.o. Box 10 01 31, 33501 Bielefeld, Germany; 2 Section of Health Equity Studies & Migration, Dept. of General Practice & Health Services Research, Heidelberg University Hospital, Im Neuenheimer Feld 130.3, 69120 Heidelberg, Germany

**Keywords:** Psychosocial functioning, Asylum seekers, Refugees, Mental Health, Healthcare

## Abstract

**Background:**

The mental health condition and healthcare needs of asylum seeking and refugee (ASR) children may go unrecognized if barriers to healthcare access exist accompanied by exclusive focus on somatic illness. We analysed the relationship between psychosocial functioning, health status and healthcare access of ASR children.

**Methods:**

During 2018, 560 ASR adults in 58 collective accommodations in Germany’s 3rd largest federal state were randomly sampled and assessed. The parent-reported Strengths and Difficulties Questionnaire (SDQ) was used to assess child psychosocial functioning. SDQ dimensions (Emotional, Conduct, Peer, Hyperactivity, Prosocial, Total) were compared by demographics (sex, age, region of origin, time since arrival, subjective social status), health status (long-lasting illness, physical limitation, pain) and healthcare access (utilization: paediatrician, specialist, dentist, psychologist, hospital/emergency department, prescribed medicines; and unmet needs: for paediatrician/specialist, reduced spending to cover healthcare cost). Age and sex-adjusted odds ratios (AOR, 95%CI-Confidence Intervals) for scoring in borderline/abnormal ranges in SDQ dimensions were estimated through logistic regression depending on children’ health status and healthcare access.

**Results:**

We analysed parents’ answers pertaining to 90 children aged 1–17 years old, 57% of which were girls and 58% with (Eastern or Western) Asian nationality. Scoring in the borderline/abnormal range of the SDQ Total Difficulties score was associated with feeling bodily pain (compared to no pain, AOR, 95%CI = 3.14, 1.21–8.10) and with an unmet need for a specialist during the previous year (4.57, 1.09–19.16). Borderline/abnormal SDQ Emotional scores were positively associated with a long-lasting illness (5.25, 1.57–17.55), physical limitation (4.28, 1.49–12.27) and bodily pain (3.00, 1.10–8.22), and negatively associated with visiting a paediatrician (0.23, 0.07–0.78), specialist (0.16, 0.04–0.69), and the emergency department (0.27, 0.08–0.96).

**Conclusion:**

Poor psychosocial functioning among ASR children is associated with somatic problems, unmet medical needs, and lower healthcare utilisation. Somatic clinical encounters with ASR should include children’ mental health symptomatology assessment, especially in those with worst physical health conditions.

**Supplementary Information:**

The online version contains supplementary material available at 10.1186/s13034-021-00411-4.

## Background

The adversities that characterize forced migration and resettlement can have a negative impact on the mental health of exposed adults and children [1, 2]. In the European Union, the mental health of asylum seeking and refugee children has been a growing source of concern, since nearly 50% of displaced ASR in 2016 were under 18 years old [3]. The difficulties entailed by migration and resettlement can impact children’s mental health and development substantially [4].

Factors affecting migrant children’s mental health include: (a) pre-migration traumatic events such as war-related violence and loss of family members; (b) peri-migration factors such as disruption of schooling, lack of security, material deprivation, food insecurity or poverty; (c) post-migration factors, such as perceived stigmatization, language barriers, frequent relocations, insecurity and lack of access to healthcare [5, 6].

According to a recent (2019) meta-analysis that included studies form all world regions, there is a great need to provide early support and promote the mental health of adult ASR and of refugee children, given prevalence estimates obtained among these groups for posttraumatic stress disorder (PTSD, 23%), depression (14%) and anxiety disorders (16%) [7].

Germany plays a relevant role as host country for asylum seekers and refugees. By the end of 2019, Germany hosted almost 1.5 million ASR [8]. Thus, the physical health, mental health and healthcare needs of ASR has been the focus of individual previous studies conducted locally [9].

Compared to German children, ASR children present higher levels of several physical illnesses, including for example dental caries [10], airway infections [11], infectious diseases[12] and skin diseases [13], for example. They are also more likely to be injured compared to resident children in Germany [14] and present higher rates of avoidable hospitalisation which suggests delayed access to primary care [15].

Regarding mental health, an earlier (2008) assessment of 55 refugee children and adolescents (aged 11–17 years old) living in Munich shelters, documented highly stressful living conditions and frequent emotional and behavioural symptoms [16]. A high rate of PTSD (30%) was also reported by a study conducted in a Munich reception camp (*Bayernkaserne*) in a sample of 986 Syrian refugee children, aged 0–14 years old [17]. Another study that explored mental health conditions of 104 ASR children in 13 Baden-Württemberg reception centres, estimated a prevalence of 19% for PTSD, and only 5% of these children received any form of therapy [18]. However, not all these studies resorted to standardized and validated instruments for assessing children mental health outcomes or psychosocial functioning, and no previous exploration was conducted at the population level, but rather using convenience or clinical samples, thus posing limits to generalization of findings to the population of ASR children in Germany.

Furthermore, the rise in ASR in the country is still not translated in the development of equitable healthcare access for these vulnerable groups. Their healthcare needs remain often unmet, particularly during the first 18 months of stay, when coverage is limited to acute and painful conditions, immunizations, preventive care and check-ups for children, services during pregnancy and childbirth, and undefined “essential services” made available as per local authorities’ consideration [19]. Furthermore, health entry screenings exclusively focus on somatic symptoms pointing to potential infectious diseases or acute health problems.

ASR children are entitled to the same care as adult ASR during the first months of their arrival. Depending on their place of residence, adult ASR need to obtain a healthcare voucher to access care and some states have introduced an electronic health card (EHC) [20]. Other necessary treatments (such as psychotherapy, for example), are granted on a case-by-case basis [21]. The healthcare voucher is obtained from local social welfare offices, and is given to the ASR’s healthcare provider of choice upon visit (commonly a general practitioner- GP). Providers issue bills to the social welfare offices who refund them. If GPs make a referral to a specialist or for hospitalization, a legal review is conducted by the social welfare office, except for emergency treatment, for which the healthcare voucher is not needed [21]. In the case of the electronic health care, they are used by ASR in the same way as healthcare vouchers, except that ASR keep the card for re-use, providers refunding is organised by the German statutory health insurance scheme, and it functions without the case-by-case reviewing for referrals. These schemes access models operating in parallel, to the access provided for regular immigrants and national citizens.

These circumstances, combined with language and geographic barriers, constitute clear barriers to healthcare access, that may contribute to the perpetuation or aggravation of poor mental health conditions that characterize ASR children. The current study was conducted as part of the RESPOND project. Details about the project, can be found at https://respond-study.org/. In brief, the project aims to generate evidence that can make healthcare structures for asylum seekers more effective, efficient, and needs-based in the long term, instead of focusing on a particular disease or health condition. First, the project aims to identify modifiable barriers to healthcare, that can be used to tailor solutions and interventions to improve existing services. These interventions are then piloted in selected settings and locations for broader implementation. So far, the project has identified a high overall health burden, including high prevalence of depressive and anxiety symptoms, and of unmet healthcare needs in adult ASR [22].

In the present study, we aimed to (i) estimate the prevalence of psychosocial symptoms among ASR children, (ii) explore the relationship between psychosocial symptoms of ASR children and somatic health status, and (iii) assess the relation between psychosocial functioning and healthcare access, including unmet needs for healthcare and healthcare utilisation.

## Methods

### Sampling, recruitment and participants

The RESPOND project is a cross-sectional, population-based assessment of asylum seekers, refugees and their children living in reception centres and regional accommodation centres in Baden-Wurttemberg, Germany [23, 24]. In Germany, ASR are initially housed in large, state reception centres, after which they can be quasi-randomly transferred into districts (NUTS-3) based on an administrative quota. They are allowed to move to independent housing after a period of 18 months or if granted refugee status, but they can stay for a longer period in the collective accommodation centre, if they are not able to find independent housing.

For the RESPOND project, a list of all accommodation centres in the state was obtained from regional authorities. Centres were then randomly selected based on a balanced sampling, selecting 58 of 1938 facilities in the state covering 1% of the 70,634 ASR registered in the state [23]. In addition, six state reception centres were purposively selected, with random sampling of 25% of rooms within each reception centre [24]. The sampling of adult ASR in the RESPOND project was conducted to meet two goals. At the level of the reception centres, sampling was conducted to obtain an equal selection probability for each person within the sampled population. At the level of accommodation centres a random sample proportional to the population was drawn, balancing on the number of refugees in the district as well as accommodation size. Further details about the sampling procedures have been previously described and the sample can be regarded as representative for the ASR population in the third largest German federal state in which the survey was conducted [22, 23]. All eligible residents of each selected accommodation unit were invited to participate. Adult ASR (18 years or older) living in these centres that could speak one of nine survey languages (English, German, Albanian, Arabic, Farsi, French, Russian, Serbian, Turkish), were eligible to participate (a total of 161 participants (6.0%) were not included due to not speaking one of the study languages).

Within each selected accommodation centre, trained teams of multi-lingual researchers approached residential rooms and invited ASR individually for participation, explaining the study objectives, the voluntary and confidential nature of participation and the anonymity of results.

Participants were asked to answer a standardized questionnaire and return it to the team personally, by post (using a prepaid envelope) or by completing the questionnaire online (a QR-code was created linking to an online version).

When first contacted, participants were also asked whether they had children (under 18 years old) living with them and were given an additional children-version module of the questionnaire to fill. They were asked to answer on behalf of one of their children, and if they had more than one, to choose the one whose last birthday was closest to the date of the survey. In total, 560 adult ASR were recruited, corresponding to 411 from accommodation centres and 149 from reception centres (response rate of 39.2%) [24]. Out of 169 distributed children’s questionnaires, 126 questionnaires were returned. A flowchart describing participants enrolement and included for analysis is shown in Additional File 1: Figure S1.

### Children’s questionnaire

The children’s questionnaire covered demographic characteristics (month and year of birth, sex, month and year of arrival to Germany) and the number of times the child has been transferred between different cities. Children’s age was categorized in three groups, from 1 to 4 years old, 5 to 9 years old and 10 to 17 years old. Country of origin was categorized according to the UN Geoscheme into Eastern Europe, Southern Europe, Western Asia, Southern Asia, Western Africa, Central Africa and Other. Time since arrival was categorized in less or more than one year and the number of times the child has been transferred between different cities was categorized as None, Once and Twice or more. The remaining questions of the children’s questionnaire were developed by the research team based on questions from the European Health Interview Survey (EHIS), the Study on the Health of Adults in Germany (DEGS) and the Study on the Health of Children and Adolescents in Germany (KiGGS) [23].

### Children’s outcomes

#### Psychosocial functioning

Children’s psychosocial functioning was measured by the parent-version of the Strengths and Difficulties Questionnaire [25]. The SDQ is a 25-item questionnaire that can be answered by parents and captures five psychosocial functioning areas (corresponding to five subscales): Emotional Symptoms, Conduct Problems, Hyperactivity, Peer Problems and Prosocial Behaviour. Each subscale is composed of five items presenting statements about children’s behaviours or emotions, with three answering options (not true, somewhat true, certainly true). Higher scores in each subscale indicate more frequent problems except for the Prosocial Behaviour. A total difficulties score is created by summing scores from all the scales except the Prosocial Behaviour scale.

For analysis, the scores for each SDQ dimension and the Total Difficulties scores were categorized in normal, borderline or abnormal range following previously published cut-off scores [26], and dichotomized as normal and borderline/abnormal (for the Total Difficulties, cut-offs were 0–13 = normal, 13–16 = borderline, 16–40 = abnormal; for the Emotional, Conduct and Peer dimensions, cut-off scores were 0–4 = normal, 4–5 = borderline, 5–10 = abnormal; for the Hyperactivity dimension cut-off scores were 0–6 = normal, 6–7 = borderline, 7–10 = abnormal; for the Prosocial dimension cut-off scores were 0–5 = abnormal, 5–6 = borderline and 6–10 = normal).

### Children’s health status

Children’s health-status was first assessed with the question “How is your child’s health in general?” with answering options: very good, good, fair, bad and very bad. These were categorized in two classes as Very good/good and Fair/bad/very bad.

Parents were asked about any long-lasting illness (for at least six months) or health problem (yes/no) of the child and to what extent has the child been limited because of a health problem in activities people usually do, in the past 12 months (answering options: severely limited, limited but not severely, not limited at all). A child’s limitation because of a health problem was coded in two categories: limited (severe or not) and not limited.

Bodily pain was assessed with the question “How much bodily pain has your child had during the past 2 weeks?” The answering options were none, very mild, mild, moderate, severe, very severe, and were categorized as “some” and “none”.

### Children healthcare access: utilization and unmet need

Parents were asked about the last time they consulted, on behalf of their child, a Paediatrician, a Specialist, a Dentist and a Psychologist, Psychotherapist or Psychiatrist: less than 12 months ago, 12 months ago or longer, don’t know, never. These answers were dichotomized as: less than 12 months and 12 months or longer/don’t know.

Also, two questions were used to assess if, during the previous 12 months, a) the child had been in hospital as an inpatient, that is overnight or longer and b) the child had been to the emergency department of a hospital. Answers were categorized as yes/no (category “don’t know” was considered a “no”).

Parents were asked if during the previous four weeks, has their child used any medicines that were prescribed by a doctor (answering options: yes/no/don’t know, categorized as yes/no, with category “don’t know considered a “no”).

An unmet need for a paediatrician was asked with the following question: “During the past 12 months, was there any time when your child really needed to consult a paediatrician (children’s doctor), but did not? (yes/no/don’t know). A similar phrasing was used to assess about an unmet need for a specialist: “During the past 12 months, was there any time when your child really needed to consult a specialist, but did not? (yes/no/don’t know).

Parents were also asked if during the previous 12 months, they reduced spending on essential needs, such as food or clothing, to be able to cover healthcare cost for their child (yes/no).

Answers to the questions about an unmet need for a paediatrician, unmet need for a specialist, and having to reduce on spending on essential needs, were also categorized as yes/no (i.e. category don’t know was considered a “no”).

### Adult questionnaire

The self-completed questionnaires covering adults’ ASR basic demographics (age, sex, marital status, nationality) and socioeconomic characteristics (educational level, income) were linked via a household identifier. The 10-rung MacArthur social scale was administered as a measure of subjective social status (SSS) [27] with reference to the country of origin and with reference to Germany, and categorized in Low (rungs 1–4), Medium (rungs 5–6) and High (7–10).

Adult questionnaires also included questions about the time since arrival to Germany and several aspects of participant’s health-status, healthcare utilization, quality of life and mental health symptoms [23].

### Participant selection, missing data, and psychometric properties

Of the 126 children cases, we excluded 36 from analysis, corresponding to 21 children one year old or younger since the SDQ might not be valid for very young children [28], and 15 children without information for the age variable. Simple mean imputation was computed for missing values by each SDQ subscale, for the remaining 90 cases (missing values ranged from 12.2% for item 16 to 25.6% for item 9). In this sample, Cronbach alphas for the Emotional Symptoms subscale was 0.615, for the Conduct Problems it was 0.530, Hyperactivity was 0.751, Peer Problems was 0.546 and Prosocial Behaviour was 0.706.

### Statistical analysis

#### Prevalence of psychosocial functioning

For each SDQ dimension, the prevalence of ASR children scoring in the normal, borderline or abnormal score range was computed, and proportions were compared with the published German normative values [26], using Chi-square goodness of fit.

### Associations between psychosocial functioning and predictor variables

To explore the distribution of the psychosocial functioning scores of ASR children, the mean (standard deviation) and the median (interquartile range, or range) of the SDQ Total Difficulties score were computed according to the categories of all demographics, social, health status and healthcare access characteristics assessed. Mann–Whitney’s U or Kruskal–Wallis’ H tests were used to compared medians across the characteristics assessed.

To assess the magnitude of associations between poor psychosocial functioning and the health status and healthcare access (utilization and unmet needs) characteristics assessed, age and sex-adjusted logistic regression models were fitted (and adjusted odds ratios, 95% Confidence Intervals—AOR, 95%CI—were estimated) between children scoring in normal vs. borderline/abnormal range scores in each SDQ dimension, entering the health status and healthcare access characteristics as independent variables. For all tests conducted, statistical significance was set at the p = 0.05 level.

### Ethical considerations

The Ethics Committee of the Medical Faculty Heidelberg approved the study protocol (reference S-516/2017).

## Results

### Sample demographic characteristics

The sample analysed was composed of 56% (n = 48) girls, 40% (36) were aged between 10 to 17 years old (Table 1). More than 58% (64) of the sample was originally from Asia and 56% (44) arrived in Germany more than a year before data collection. 81% (73) were in a regional accommodation centre, and 44% (35) of the sample has been transferred between different cities in Germany two or more times. Of the parents, 42% (27) declared having had a “low” subjective social status in the country of origin and this proportion was 76% (47) when referring to their subjective social status in Germany. There were no statistically significant differences observed in the SDQ Total Difficulties median score according to the characteristics listed in Table 1.

**Table 1:** Sample characteristics (excluding ages 0–1 years old) according to strengths and difficulties questionnaire scores (SDQ)

		SDQ Total Difficulties score	
	n (%)	mean (sd)	Median (IQR or range§)
Sex (missings = 5)			
Male	37 (43.5)	12.56 (7.32)	12.00 (8.13)
Female	48 (56.5)	11.87 (5.57)	12.00 (5.45)
Age categories			
1–4 years old	32 (35.6)	11.19 (5.47)	10.58 (7.92)
5–9 years old	22 (24.4)	13.57 (5.75)	13.17 (7.82)
10–17 years old	36 (40.0)	12.01 (7.10)	12.00 (6.00)
Region of origin (missings = 10)			
Eastern Europe	5 (6.3)	13.13 (4.77)	13.00 (7.67)
Southern Europe	3 (3.3)	14.00 (3.50)	13.00 (6.78§)
Western Asia	31 (34.4)	11.74 (5.77)	10.58 (5.58)
Southern Asia	22 (24.4)	11.06 (5.69)	12.00 (7.93)
Western Africa	11 (12.2)	10.13 (5.74)	10.58 (11.27)
Central Africa	2 (2.2)	11.96 (1.95)	11.96 (2.75§)
Other	6 (6.7)	14.24 (8.64)	14.50 (13.82)
Time since arrival (missings = 11)			
Less than a year	35 (44.3)	13.16 (6.47)	11.43 (8.33)
More than a year	44 (55.7)	11.15 (6.10)	11.79 (6.00)
Accommodation type			
GU	73 (81.1)	11.82 (5.79)	11.58 (5.95)
LEA	17 (18.9)	13.28 (7.99)	13.00 (8.24)
How many times has your child been transferred between different cities or towns in Germany? (missings = 10)			
None	22 (27.5)	12.02 (5.96)	11.29 (7.93)
Once	23 (28.7)	14.74 (6.73)	14.00 (6.89)
Twice or more	35 (43.8)	11.05 (5.89)	10.58 (8.33)
Parental Subjective Social Status in country of origin (missings = 26)			
Low	27 (42.2)	12.57 (7.00)	12.00 (7.79)
Medium	19 (21.1)	13.27 (5.64)	12.00 (7.89)
High	18 (20.0)	10.83 (3.67)	11.29 (5.50)
Parental Subjective Social Status in Germany (missings = 28)			
Low	47 (75.8)	12.88 (6.07)	13.00 (7.58)
Medium	12 (19.4)	11.63 (5.69)	11.35 (4.25)
High	3 (4.8)	10.00 (3.33)	10.00 (3.33)

*SDQ* Strengths and Difficulties Questionnaires, *sd* standard deviation, *IQR* Interquartile Range, *GU* *Gemeinschaftsunterkünfte (Shared accommodation)*, *LEA Aufnahmeeinrichtung (Reception centre)*

### Prevalence of psychosocial function

Compared with the normative values published for children in Germany [26], a higher proportion of participants answering in the abnormal range score was observed for the Total Difficulties score (22 vs. 10%), for the Emotional symptoms dimension score (33 vs. 8%) and for the Peer Problems dimension score (26 vs. 7%), Table 2.

**Table 2:** Normal, Borderline and Abnormal range scores in the Strengths and Difficulties Questionnaire in the sample and in Germany [26]*

	Strengths and Difficulties Questionnaire							
	Normal range		Borderline range		Abnormal range		Cronbach Alpha	
	Sample (n = 90, ages 1-17 years)	Germany* (n = 930, ages 6–16 years)	Sample	Germany	Sample	Germany	Sample	Germany
	n (%)	%	n (%)	%	n (%)	%		
Total Difficulties score§	52 (57.8)	81.6	18 (20.0)	8.4	20 (22.2)	10.0	0.75¥	0.82
Emotional symptoms§	56 (62.2)	86.0	4 (4.4)	6.3	30 (33.3)	7.7	0.73	0.66
Conduct problems	80 (88.9)	84.7	2 (2.2)	8.7	8 (8.9)	6.6	0.34	0.60
Peer problems§	55 (61.1)	86.7	12 (13.3)	6.3	23 (25.6)	7.0	0.18	0.58
Hyperactivity/Inattention§	68 (75.6)	85.3	14 (15.6)	4.9	8 (8.9)	9.8	0.48	0.76
Prosocial behaviour	80 (88.9)	84.4	2 (2.2)	8.5	8 (8.9)	7.1	0.55	0.68

^*^Woerner et al. (2002)

¥ item 22 removed (reliability computed for 19 items in the Total Difficulties score)

§Proportions are significantly different across the 3 groups (normal, borderline, abnormal, p < 0.05 for chi-square goodness of fit) between the sample and Germany for the Total Difficulties score, the Emotional symptoms, Peer problems and Hyperactivity dimensions

### Description of children health status and healthcare access (utilization and unmet needs)

More than 72% (62) of parents referred to their child’s general health status as very good or good (Table 3), about 26% (22) declared that the child had a long-lasting illness or health problem (a condition lasting six months or more), and 37% (29) said the child has been limited because of a health problem in the past 12 months. For 48% (42) of children, their parents reported that they had some bodily pain during the previous year.

**Table 3:** Health status and healthcare access characteristics according to the Strengths and Difficulties questionnaire scores (SDQ)

			SDQ Total Difficulties score	
		n (%)	mean (sd)	Median (IQR)
Health status				
General Health	Very Good/Good	62 (72.1)	11.48 (5.24)	11.58 (5.76)
	Fair/Bad/Very Bad	24 (27.9)	13.94 (8.53)	13.00 (11.25)
Any long-lasting illness (child) (missings = 5)	Yes	22 (25.9)	15.33 (7.59)	13.00 (9.66)
	No	63 (74.1)	11.01 (5.57)	11.00 (6.00)*
Has the child been limited because of a health problem? (missings = 12)	limited (severe or not)	29 (37.2)	14.66 (7.77)	13.00 (8.92)
	not limited	49 (62.8)	11.04 (5.24)	11.11 (6.50)*
Bodily pain during past year? (missings = 3)	None	45 (51.7)	10.38 (5.51)	10.58 (6.83)
	Some	42 (48.3)	13.74 (6.64)	13.00 (7.34)*
Healthcare utilization				
Last time visited a Paediatrician (missings = 17)	Less than 12 months	45 (61.6)	13.00 (6.30)	12.00 (6.07)
	Never/12 months or longer/Don´t know	28 (38.4)	10.64 (6.92)	11.00 (10.54)
Last time visited a Specialist (missings = 33)	Less than 12 months	16 (28.1)	15.48 (8.60)	13.67 (11.44)
	Never/12 months or longer/Don´t know	41 (71.9)	10.81 (5.63)	11.00 (6.50)
Last time visited a Dentist (missings = 24)	Less than 12 months	26 (39.4)	13.06 (5.90)	12.50 (8.03)
	Never/12 months or longer/Don´t know	40 (60.6)	11.80 (7.23)	11.79 (8.39)
Last time visited a Psychologist, psychotherapist or psychiatrist (missings = 33)	Less than 12 months	5 (8.8)	23.07 (8.21)	21.33 (14.67)
	Never/12 months or longer/Don´t know	52 (91.2)	10.93 (5.67)	11.06 (6.00)*
During past 4 weeks has your child used any medicines prescribed by a doctor? (missings = 5)	Yes	43 (50.6)	12.83 (6.65)	13.00 (6.79)
	No	42 (49.4)	11.35 (6.08)	11.06 (5.93)
Has your child been in hospital inpatient last 12 months? (missings = 5)	Yes	14 (16.5)	13.86 (9.08)	11.71 (10.36)
	No/Don’t know	71 (83.5)	12.01 (5.59)	12.00 (6.58)
Emergency department visit last 12 months (missings = 7)	Yes	16 (19.3)	14.16 (7.65)	12.50 (7.86)
	No/Don’t know	67 (80.7)	11.75 (6.09)	12.00 (7.00)
Unmet medical need				
Unmet need for a paediatrician during past year? (missings = 15)	Yes	18 (24.0)	14.41 (6.99)	13.00 (8.25)
	No	57 (76.0)	11.64 (5.84)	11.58 (6.79)
Unmet need for a specialist during the past year? (missings = 14)	Yes	13 (24.1)	16.07 (6.64)	17.33 (8.53)
	No	63 (75.9)	11.62 (6.21)	11.43 (6.00)*
Reduction on spending on essential needs, such as food or clothing, to be able to cover healthcare costs for the child? (missings = 9)	Yes	18 (22.8)	14.08 (7.33)	13.67 (8.40)
	No	61 (77.2)	11.75 (6.05)	12.00 (6.37)

*SDQ *Strengths and Difficulties Questionnaires, *sd* standard deviation, *IQR* Interquartile Range

*p < 0.05 for the Kruskal–Wallis or Mann–Whitney test comparing median scores

Almost 62% (45) declared having visited a Paediatrician in the previous 12 months and 16 participants reported a visit to a Specialist (for the child) in the previous 12 months. Only 5 parents (almost 9% of respondents to this question) referred having visited a Psychologist, Psychotherapist or Psychiatrist in the previous 12 months. Regarding unmet medical needs, 24% (18) of parents reported that during the previous 12 months, there was a time when their child really needed to consult a Paediatrician but did not. The same proportion of unmet needs were reported for specialist visits. 23% (18) of parents reported that they had to reduce their spending on essential needs, such as food or clothing, to be able to cover healthcare cost for their child in the previous 12 months.

During the 4 weeks prior to the interview, nearly half (43) of participants declared that their child used medicines prescribed by a doctor. Also, 16% (14) said their child stayed in the hospital as an inpatient during the past 12 months and 19% (16) reported a visit to the Emergency department for the same period.

### Description of psychosocial functioning by health status and healthcare access

The SDQ Total Difficulties median score was higher, suggesting more frequent symptoms of poor psychosocial functioning and showing a statistically significant difference: for children with a long-lasting illness, compared with those who did not declare such condition; for children who were physically limited compared to children not limited; for children who felt some bodily pain during the past year compared with those who did not feel pain; for those whose parents declared an unmet need for a Specialist during the past year compared to those who did not report it; and for those whose parents reported a visit to a Psychologist, Psychotherapist or Psychiatrist in less than 12 months, compared to those who declared never having visited any of these professionals, for more than 12 months or did not know (Table 3).

### Association between psychosocial functioning and health status

Scoring above 13 points (borderline or abnormal range) in the SDQ Total Difficulties score was associated with feeling some bodily pain during the previous year (compared to feeling no pain, AOR, 95%CI = 3.14, 1.21–8.10), independently of age and sex of children, Table 4.

**Table 4:** Associations between the Strengths and Difficulties Questionnaire scores (SDQ—normal vs. borderline or abnormal range) and the health status and healthcare utilization characteristics

		SDQ Total > 13 Borderline or Abnormal, n = 38		SDQ emotional > 4, n = 34		SDQ conduct > 4, n = 10	
		OR (95% CI)	AOR (95% CI)	OR (95% CI)	AOR (95% CI)	OR (95% CI)	AOR (95% CI)
Health status							
General Health	Fair / Bad / Very Bad (Ref = Very Good / Good)	1.75 (0.68–4.52)	1.94 (0.69–5.40)	2.31 (0.88–6.03)	2.00 (0.69–5.76)	4.83 (1.23–19.04) *	4.59 (1.09–19.37) *
Any long-lasting illness (child) (missings = 5)	Yes (Ref = No)	2.35 (0.87–6.32)	2.74 (0.88–8.59)	6.67 (2.25–19.75) *	5.25 (1.57–17.55) *	3.41 (0.88–13.19)	3.97 (0.78–20.26)
Has the child been limited because of a health problem? (missings = 12)	limited (severe or not) (Ref = not limited)	2.44 (0.95–6.25)	2.67 (0.96–7.42)	4.75 (1.77–12.72) *	4.28 (1.49–12.27) *	1.83 (0.48–6.97)	1.56 (0.39–6.19)
Bodily pain during past year? (missings = 3)	Some (Ref = None)	2.98 (1.23–7.23)*	3.14 (1.21–8.10)*	2.24 (0.92–5.42)	3.00 (1.10–8.22) *	10.35 (1.24–86.81) *	10.30 (1.21–87.56) *
Healthcare utilization							
Last time visited a Paediatrician (missings = 17)	Never/12 months or longer/Don´t know (Ref = Less than 12 months)	0.58 (0.22–1.53)	0.43 (0.15–1.27)	0.25 (0.08–0.77) *	0.23 (0.07–0.78) *	1.33 (0.33–5.46)	1.53 (0.35–6.60)
Last time visited a Specialist (missings = 33)	Never/12 months or longer/Don´t know (Ref = Less than 12 months)	0.35 (0.11–1.14)	0.40 (0.11–1.37)	0.22 (0.07–0.75) *	0.16 (0.04–0.69) *	0.42 (0.10–1.81)	0.52 (0.11–2.53)
Last time visited a Dentist (missings = 24)	Never/12 months or longer/Don´t know (Ref = Less than 12 months)	0.82 (0.30–2.20)	0.85 (0.27–2.69)	0.56 (0.20–1.55)	1.01 (0.31–3.27)	1.63 (0.38–6.96)	1.89 (0.39–9.12)
Last time visited a Psychologist, psychotherapist or psychiatrist (missings = 33)	Never/12 months or longer/Don´t know (Ref = Less than 12 months)	–	–	0.11 (0.01–1.07)	0.11 (0.01–1.33)	0.20 (0.03–1.42)	0.12 (0.08–1.65)
During past 4 weeks has your child used any medicines prescribed by a doctor? (missings = 5)	No (Ref = Yes)	0.44 (0.18–1.05)	0.38 (0.14–1.00)	0.77 (0.32–1.85)	0.38 (0.13–1.10)	1.03 (0.27–3.84)	0.94 (0.23–3.89)
Has your child been in hospital inpatient last 12 months? (missings = 5)	No/Don’t know (Ref = Yes)	1.09 (0.34–3.48)	1.29 (0.39–4.33)	0.87 (0.27–2.77)	0.98 (0.28–3.49)	0.40 (0.09–1.79)	0.48 (0.10–2.22)
Emergency department visit last 12 months (missings = 7)	No/Don’t know (Ref = Yes)	0.76 (0.26–2.28)	0.72 (0.23–2.20)	0.34 (0.11–1.04)	0.27 (0.08–0.96) *	0.95 (0.18–4.97)	1.00 (0.19–5.33)
Unmet medical need							
Unmet need for a paediatrician during past year? (missings = 15)	Yes (Ref = No)	2.50 (0.84–7.42)	2.11 (0.66–6.82)	1.37 (0.47–4.02)	1.29 (0.38–4.44)	0.89 (0.17–4.74)	0.63 (0.11–3.71)
Unmet need for a specialist during the past year? (missings = 14)	Yes (Ref = No)	5.42 (1.35–21.68) *	4.57 (1.09–19.16) *	0.95 (0.28–3.24)	1.21 (0.32–4.64)	2.85 (0.61–13.30)	3.16 (0.62–16.00)
Reduction on spending on essential needs, such as food or clothing, to be able to cover healthcare costs for the child? (missings = 9)	Yes (Ref = No)	3.08 (1.02–9.32) *	2.68 (0.84–8.50)	1.93 (0.67–5.57)	1.78 (0.55–5.72)	3.20 (0.76–13.50)	2.97 (0.66–13.39)

		SDQ peer > 4, n = 35		SDQ hyperactive > 6, n = 22		SDQ Prosocial < 6, n = 10	
		OR (95% CI)	AOR (95% CI)	OR (95% CI)	AOR (95% CI)	OR (95% CI)	AOR (95% CI)
Health status							
General Health	Fair / Bad / Very Bad (Ref = Very Good / Good)	2.15 (0.83–5.59)	2.20 (0.77–6.24)	1.29 (0.45–3.71)	1.75 (0.56–5.41)	1.12 (0.27–4.75)	1.45 (0.29–7.22)
Any long-lasting illness (child) (missings = 5)	Yes (Ref = No)	4.96 (1.74–14.13) *	5.14 (1.53–17.21) *	0.86 (0.27–2.72)	1.22 (0.34–4.40)	1.26 (0.30–5.38)	2.00 (0.34–11.99)
Has the child been limited because of a health problem? (missings = 12)	limited (severe or not) (Ref = not limited)	3.54 (1.35–9.29) *	3.70 (1.31–10.42) *	0.65 (0.22–1.94)	0.75 (0.24–2.36)	0.83 (0.19–3.59)	0.86 (0.17–4.31)
Bodily pain during past year? (missings = 3)	Some (Ref = None)	1.23 (0.52–2.93)	1.18 (0.46–3.02)	2.07 (0.76–5.67)	2.04 (0.70–5.93)	1.08 (0.29–4.04)	0.69 (0.16–3.06)
Healthcare utilization							
Last time visited a Paediatrician (missings = 17)	Never/12 months or longer/Don´t know (Ref = Less than 12 months)	0.55 (0.20–1.50)	0.41 (0.13–1.26)	1.30 (0.46–3.66)	1.10 (0.37–3.27)	2.23 (0.54–9.13)	1.93 (0.37–9.93)
Last time visited a Specialist (missings = 33)	Never/12 months or longer/Don´t know (Ref = Less than 12 months)	0.17 (0.05–0.59) *	0.14 (0.03–0.58) *	0.54 (0.16–1.85)	0.50 (0.14–1.87)	0.47 (0.09–2.38)	0.32 (0.04–2.44)
Last time visited a Dentist (missings = 24)	Never/12 months or longer/Don´t know (Ref = Less than 12 months)	0.28 (0.10–0.79) *	0.29 (0.09–0.96) *	1.61 (0.52–4.95)	0.92 (0.25–3.45)	5.30 (0.61–45.93)	5.96 (0.51–70.20)
Last time visited a Psychologist, psychotherapist or psychiatrist (missings = 33)	Never/12 months or longer/Don´t know (Ref = Less than 12 months)	–	–	0.25 (0.04–1.63)	0.16 (0.02–1.33)	0.16 (0.02–1.19)	0.07 (0.00–1.36)
During past 4 weeks has your child used any medicines prescribed by a doctor? (missings = 5)	No (Ref = Yes)	1.04 (0.44–2.48)	0.85 (0.32–2.23)	0.81 (0.31–2.14)	1.03 (0.36–2.94)	0.26 (0.05–1.32)	0.28 (0.05–1.73)
Has your child been in hospital inpatient last 12 months? (missings = 5)	No/Don’t know (Ref = Yes)	1.32 (0.40–4.33)	1.62 (0.46–5.75)	1.34 (0.34–5.33)	1.28 (0.31–5.36)	0.40 (0.09–1.79)	0.45 (0.09–2.33)
Emergency department visit last 12 months (missings = 7)	No/Don’t know (Ref = Yes)	1.20 (0.39–3.68)	1.09 (0.34–3.51)	1.02 (0.29–3.59)	1.02 (0.28–3.63)	0.51 (0.12–2.22)	0.41 (0.08–2.06)
Unmet medical need							
Unmet need for a paediatrician during past year? (missings = 15)	Yes (Ref = No)	1.59 (0.55–4.62)	1.26 (0.39–4.09)	1.54 (0.49–4.85)	1.42 (0.38–5.34)	0.89 (0.17–4.74)	0.30 (0.03–2.99)
Unmet need for a specialist during the past year? (missings = 14)	Yes (Ref = No)	6.21 (1.55–24.95) *	6.02 (1.36–26.74) *	2.74 (0.80–9.43)	2.15 (0.57–8.13)	4.43 (0.86–22.82)	2.24 (0.31–16.08)
Reduction on spending on essential needs. such as food or clothing. to be able to cover healthcare costs for the child? (missings = 9)	Yes (Ref = No)	1.54 (0.54–4.44)	1.23 (0.39–3.86)	1.68 (0.53–5.29)	1.97 (0.57–6.84)	3.20 (0.76–13.50)	2.66 (0.53–13.40)

OR (95%CI): Odds ratio (95% Confidence intervals); AOR: Adjusted odds ratio – adjusted for sex and age; SDQ – Strengths and Difficulties Questionnaire; Ref = Reference category.. *Estimate statistically significant (p < 0.05)

Scoring above 4 points in the SDQ Emotional symptoms dimension (borderline or abnormal range), was associated with the presence of a long-lasting illness (AOR, 95%CI = 5.25, 1.57–17.55), with having been physically limited because of a health problem (AOR, 95%CI = 4.28, 1.49–12.27), and with bodily pain (AOR, 95%CI = 3.00, 1.10–8.22).

For the Conduct Problems dimension, scoring within or above the borderline range was associated with a poor general health status (AOR, 95%CI = 4.59, 1.09–19.37), and with some bodily pain (AOR, 95%CI = 1.21–87.56).

A borderline or abnormal score in the Peer problems dimension was associated with the presence of a long-lasting illness (AOR, 95%CI = 5.14, 1.53–17.21) and with a physical limitation because of a health problem (AOR, 95%CI = 3.70, 1.31–10.42).

### Associations between psychosocial functioning and healthcare access (utilization and unmet need)

A significant inverse association was noted between a high score in the SDQ Emotional symptoms dimension and having visited a Paediatrician (AOR, 95%CI = 0.23, 0.07–0.78), having visited a Specialist (AOR, 95%CI = 0.16, 0.04–0.69), and attending the emergency department during the last 12 months (AOR, 95%CI = 0.27, 0.08–0.96), Table 4.

Lower odds of scoring high in the Peer problems dimension were noted for those who declared not having visited a Specialist (AOR, 95%CI = 0.14, 0.03–0.58) or a dentist (AOR, 95%CI = 0.29, 0.09–0.96) in the previous 12 months compared to those who had visited.

Scoring above 13 points (borderline or abnormal range) in the SDQ Total Difficulties score was associated with having an unmet need for a Specialist during the previous year (AOR, 95%CI = 4.57, 1.09–19.16).

A borderline or abnormal score in the Peer problems dimension was associated with an unmet need for a Specialist during the previous year (AOR, 95%CI = 6.02, 1.36–26.74).

Statistically significant associations were not observed between a borderline or abnormal score in the Hyperactivity and Prosocial behaviour scales, and the health status and healthcare access characteristics explored.

## Discussion

This study described the associations between the psychosocial functioning dimensions of asylum seeking and refugee children with their health status and reported access to healthcare, including unmet medical needs and utilization of healthcare services. The results suggest that poorer psychosocial functioning is associated with bodily pain, with the presence of a long-lasting illness, with physical limitation and with an unmet need for a Specialist. The results also suggest that previous visits to healthcare services are inversely associated with psychosocial functioning.

This may mean that somatic health complaints identified during clinical encounters with ASR children might be indicative of the need to assess (poor) mental health symptoms, which may otherwise go unnoticed. It is likely that poor psychosocial function or mental health problems of ASR children are overlooked by healthcare services, if no outreach or population-based assessments are conducted within these groups. The identification of somatic health complaints (as identified in this study), should be thoroughly assessed as they seem to be congruently associated with mental health problems among these children.

### Interpretation of findings

The overall SDQ mean scores obtained suggest that children of ASR assessed as part of the RESPOND project have poorer mental health symptoms, when compared to the German normative values for children [26, 29]. A previous analysis of the SDQ to compare German native to immigrant reports (of Turkish and Russian origin), suggested that a direct comparison between immigrant and German parental-reports might not be adequate, but the psychometric analysis revealed that the instrument is reliable for screening mental health problems [30]. The SDQ has also been used with ASR in the context of the Syrian crisis and proven to be a feasible tool [31].

The link between poor physical health (bodily pain, long-lasting illness, physical limitation) and mental health outcomes observed in this study is congruent with previous research documenting poorer health outcomes among migrant children compared with non-migrants. European paediatricians, for example, perceive migrant children as having worse general health status, and chronic conditions are the most frequent health problems that these specialists identify [32]. Overall, this suggests that questioning about such somatic conditions, which is often faster and easier compared to lengthier and more sensitive tools (such as the SDQ), could indicate the need to further explore mental health problems that may be concomitantly present in ASR children.

We observed that the presence of a long-lasting illness, pain, worse general health-status and a physical limitation, were associated with (poorer scores in) the SDQ conduct and peer dimensions. Since these dimensions are linked with children’ social interactions, this result suggests that children’ physical health problems might have a greater impact in their social interactions with others, than in other dimensions of their psychosocial functioning. This may be further aggravated by all the processes characterizing resettlement, such as a new language, several re-locations and living in temporary accommodation. A higher score in the SDQ emotional dimension was associated with the presence of pain and with a long-lasting illness. This may suggest an association between psychological internalization processes (as opposed to externalization) and physical problems among ASR children. This is also in line with previous suggestions that refugee children might resort to a learned passivity and invisibility conduct style, which can, in turn, be reinforced by their parents, particularly when the family residence status is yet uncertain [33].Overall, ASR children’s poor psychosocial functioning, in accumulation with a cluster of known disadvantaged experiences, namely poor socioeconomic circumstances, experience of violent and traumatic events and a long-lasting or chronic condition, represents a serious risk factor for their normal development [34].

The association found between children’s poor psychosocial functioning and unmet needs for certain healthcare services is also in line with previous research [35]. A recent review exploring the reasons why levels of mental health help-seeking in ASR are low, found that stigma, financial strain, language proficiency, unstable accommodation, lack of knowledge on how to access services, lack of trust and concerns about confidentiality are amongst the main barriers for accessing healthcare [36].

A qualitative exploration conducted among Ethiopian refugees in Norway also documented that lack of information and perception of limited access to the healthcare system are among the main factors for refraining from seeking care and ability to navigate the healthcare system [37]. Syrian ASR in Switzerland face similar barriers to mental health care, related to language, lack of awareness and stigma, among others [38]. Although the provision of information on how to use the healthcare system and which services are available to ASR is generically provided in most reception centres, such information may not be received as expected and the effectiveness of such communication may need improvement, including participatory approaches that consider ASR’s needs [39].

In the current analysis, we did not explore the declared or perceived reasons for unmet medical needs. However, most of these reasons have been previously studied, related to the adaptations needed for healthcare provision since the rise in ASR entering the country since 2015 [40, 41]. Differences in regional restrictions and models of healthcare entitlement (healthcare voucher or electronic health card), that might have been attributed to them upon arrival and random assignment to a specific municipality [42], add to the likelihood of running into barriers.

We did not find a significant difference in the SDQ scores according to the time since arrival declared by participants, however, the specific effect of time elapsed until eHealth card attribution and joining a statutory health insurance scheme on healthcare services utilization was not explored in this study, and should be the focus of future research [42].

Although ASR children seem to score more frequently in a borderline or abnormal range in the Hyperactivity dimension of the SDQ, compared to non-migrant children, this subscale did not show any significant association with the health status and healthcare characteristics studied. However, the point estimates obtained suggest the same trend found for the remaining SDQ subscales. For example, children who had never visited a psychologist, psychotherapist or psychiatrist in the previous year or did so more than 12 months ago, showed an AOR of 0.16 for scoring in the borderline or abnormal range of the hyperactivity subscale, when compared with children who did make such a visit in the previous year. Children scoring higher in this subscale also showed higher odds for bodily pain (AOR = 2.04) and for unmet need for a specialist (AOR = 2.15). The absence of statistically significant associations for this subscale may be due to lack of statistical power.

### Recommendations for healthcare

Restrictive policies and healthcare barriers might have a negative impact on ASR children’s health. The current results suggest that visiting healthcare services during the previous year is associated with better psychosocial adjustment compared with not visiting them. Therefore, the provision of well-informed health services directed to children are needed, as has been suggested previously [35].

Despite the efficacy and acceptability of psychosocial interventions compared to control conditions to reduce mental health problems in ASR groups [6], there is no specific protocol to screen and provide care for child mental health suffering, thus prevalence rates will potentially remain high among these vulnerable groups, if nothing is done [43]. Given the link found between somatic health complaints and poor psychosocial functioning, the identification of easily measurable somatic conditions among ASR children could function as a proxy indicator for compromised mental health, and would facilitate the implementation of structured, wider assessments, by informing about those individuals whose mental health condition should be the focus of further evaluation.

### Strengths and limitations

A major strength of this study lies in the sampling procedure implemented to maximize representativeness and obtain a comprehensive coverage of ASR residing in Baden-Wurttemberg, the 3^rd^ largest German state. All adults with children were asked to fill the children-related questionnaire. Despite a rigorous sampling strategy, a sampling bias cannot be completely ruled out, which might have influenced the association estimates documented. For example, less educated ASR might have not filled the questionnaire: since a lower educational level is associated with poorer health outcomes, this may mean that our estimates would be underestimated, and worst outcomes would have been observed. Nevertheless, the sample’s characteristics have been previously described [23], and the sampling strategy allowed the estimation of reliable indicators for ASR health.

By the end of 2017 (when RESPOND recruitment started), there were 970,357 refugees and 429,265 asylum seekers in Germany, according to UNHCR statistics (https://www.unhcr.org/refugee-statistics-uat/download/?url=EQ07fq), and 416,195 of these were children under the age of 18 years (https://www.unhcr.org/refugee-statistics-uat/download/?url=3LlO6v). To retain feasibility and quality, and minimize the burden of answering on behalf of many children, questionnaires were delivered to adult ASR who had children under 18 years living with them and were asked to complete the questionnaire for only one child (which last had its birthday). This means, however, that it is not possible to make any judgment about the representativeness of the included sample in relation to the ASR population of children residing in Germany. The sampling frame was tailored to adult populations. Nonetheless, the sample analysed was proportionally close to children ASR residing in Germany in 2017 (e.g. 35.6% of children aged 1–4 years in the sample vs. 32.5% of total ASR children aged up to 4 years in Germany in 2017; 43.5% boys and 56.5% girls in sample vs. 44.4% ASR girls and 55.6% ASR boys in Germany in 2017 – shown in Additional file 1: Table S1).

A potential for residual confounding cannot be ruled out, since several influencing factors for physical conditions, psychosocial functioning and healthcare utilisation were not measured or analysed, thus results should be cautiously interpreted.

This study relied on parents’ self-reports of the health characteristics of their children, including their psychosocial functioning. The parent-version of the SDQ has been used with success in the assessment of very young children (from 2 years of age) [44]. However, we cannot rule out that parents provided socially desirable answers in relation to their young children, including a tendency to under-report children’s symptoms, or to give higher scores for their children symptomatology, particularly if suffering from psychological distress [45].

The parent SDQ is widely used to assess children and adolescent psychosocial functioning and has been validated for the German population and used in large nationally representative surveys [29] as well as in children refugee populations in Germany [46]. SDQ scores have been found to closely predict the prevalence of clinician-rated child mental disorders [47]. However, poor cross-informant discriminant validity has also been reported between the emotional and peer subscales and between the behavioural, hyperactivity and prosocial subscales. This suggests that in “low-risk” samples, the five sub-scales of the SDQ may not provide an ideal distinction between these different dimensions of child mental health [48] (thus suggesting the use of the total scale score or its aggregated internalizing/externalizing subscales).

## Conclusions

Several previous studies have documented the burden of mental health problems among ASR, and particularly, how such needs are frequently not met [49]. This study adds contextual knowledge to the bulk of these results, showing that children presenting poor physical health conditions and declared unmet healthcare needs in Germany could be prioritized in the existent health screening conducted among ASR groups. Furthermore, the association found between physical or somatic health complaints and poor psychosocial functioning suggests that both health dimensions should be addressed concomitantly in population-based assessments designed for ASR groups. This would enable wider health screening efforts and reduce the chances of missing important mental health problems among ASR children, which can seriously endanger their normal development.

## Supplementary Information

**Additional file 1: Figure S1**. Flowchart describing participants enrolment and included for analysis. **Table S1**. Age and sex of sampled children and ASR children in Germany, 2017.

## Data Availability

Data are available from the authors upon reasonable request.

## Abbreviations

ASR: Asylum Seekers and Refugees
SDQ: Strengths and Difficulties Questionnaire
AOR: Adjusted Odds Ratio
CI: Confidence Interval
PTSD: Posttraumatic Stress Disorder
DEGS: Study on the Health of Adults in Germany
EHIS: European Health Interview Survey
KiGGS: Study on the Health of Children and Adolescents in Germany
SSS: Subjective Social Status
