# Supplementary material for: Association between psychosocial functioning, health status and healthcare access of asylum seekers and refugee children: a population-based cross-sectional study in a German federal state
Source: Child Adolesc Psychiatry Ment Health. 2021 Oct 12;15:59. doi: 10.1186/s13034-021-00411-4 (PMC8513294; doi:10.1186/s13034-021-00411-4)
Supplement: Supplementary file 1 — Additional file 1: Figure S1. Flowchart describing participants enrolment and included for analysis. Table S1. Age and sex of sampled children and ASR children in Germany, 2017. [file 13034_2021_411_MOESM1_ESM.docx]

Supplementary material

**Total residents in selected accommodation facilities or rooms**:

Reception centres: n = 826

Accommodation centres: n = 1843

Total N: 2669 (100%)

**Residents eligible for participation**

n = 1429 (53.5%)

**Residents not eligible for participation**

Due to being underage n = 1079 (40.4%)

Due to not speaking one of the study languages n = 161 (6.0%)

**Eligible adult individuals contacted**

n = 1201 (45.0%)

**Non-participation**

Refusal at outset n = 35 (1.3%)

Questionnaire not returned/empty n = 606 (22.7%)

**Adult individuals recruited**

n = 560 (21.0%)

**Children questionnaires distributed**

n = 169

**Included in analysis**

n = 90

**Non-participation**

Children questionnaire not returned/empty n = 43

Excluded from analysis:

Aged 1 year or younger n = 21

Missing age n = 15

Figure 1S. Flowchart describing participants enrolment and included for analysis

Table S1. Age and sex of sampled children and ASR children in Germany, 2017.

|  | **Sample** | |  | **UNHCR statistics 2017*** | |
| --- | --- | --- | --- | --- | --- |
|  |  | **n (%)** |  |  | **n (%)** |
| **Sex** | **Male** | 37 (43.5) |  | **Male** | 231406 (55.6) |
|  | **Female** | 48 (56.5) |  | **Female** | 184789 (44.4) |
| **Age (in years)** | **1 to 4** | 32 (35.6) |  | **0 to 4** | 135121 (32.5) |
|  | **5 to 9** | 22 (24.4) |  | **5 to 11** | 159557 (38.3) |
|  | **10 to 17** | 36 (40.0) |  | **12 to 17** | 121517 (29.2) |

*available at https://www.unhcr.org/refugee-statistics-uat/download/?url=3LlO6v
